# Revised microbial and photochemical triple-oxygen isotope effects improve marine gross oxygen production estimates

**DOI:** 10.1093/pnasnexus/pgac233

**Published:** 2022-10-12

**Authors:** Kevin M Sutherland, David T Johnston, Jordon D Hemingway, Scott D Wankel, Collin P Ward

**Affiliations:** Department of Earth and Planetary Sciences, Harvard University, Cambridge, MA 02138, USA; Department of Earth and Planetary Sciences, Harvard University, Cambridge, MA 02138, USA; ETH Zürich, Geological Institute, Department of Earth Sciences, Zürich 8092, Switzerland; Department of Marine Chemistry and Geochemistry, Woods Hole Oceanographic Institution, Woods Hole, MA 02543, USA; Department of Marine Chemistry and Geochemistry, Woods Hole Oceanographic Institution, Woods Hole, MA 02543, USA

**Keywords:** gross primary productivity, gross oxygen production, triple-oxygen isotopes, respiration, photochemical oxidation

## Abstract

The biogeochemical fluxes that cycle oxygen (O_2_) play a critical role in regulating Earth’s climate and habitability. Triple-oxygen isotope (TOI) compositions of marine dissolved O_2_ are considered a robust tool for tracing oxygen cycling and quantifying gross photosynthetic O_2_ production. This method assumes that photosynthesis, microbial respiration, and gas exchange with the atmosphere are the primary influences on dissolved O_2_ content, and that they have predictable, fixed isotope effects. Despite its widespread use, there are major elements of this approach that remain uncharacterized, including the TOI dynamics of respiration by marine heterotrophic bacteria and abiotic O_2_ sinks such as the photochemical oxidation of dissolved organic carbon (DOC). Here, we report the TOI fractionation for O_2_ utilization by two model marine heterotrophs and by abiotic photo-oxidation of representative terrestrial and coastal marine DOC. We demonstrate that TOI slopes associated with these processes span a significant range of the mass-dependent domain (λ = 0.499 to 0.521). A sensitivity analysis reveals that even under moderate productivity and photo-oxidation scenarios, true gross oxygen production may deviate from previous estimates by more than 20% in either direction. By considering a broader suite of oxygen cycle reactions, our findings challenge current gross oxygen production estimates and highlight several paths forward to better understanding the marine oxygen and carbon cycles.

Significance Statement The oxygen isotope composition (abundance of ^17^O and ^18^O relative to ^16^O) of marine dissolved oxygen has been used for decades to fingerprint biogeochemical oxygen cycling and to estimate gross photosynthesis. However, the accuracy of this method relies heavily on our understanding of how respiration discriminates against different isotopes of oxygen. Additionally, there exist quantitatively significant nonmicrobial sinks, such as photochemical oxidation of dissolved organic carbon, that have unknown effects on this important oceanographic tool. Here, we demonstrate that the oxygen isotope fingerprints associated with model marine heterotrophs and dissolved organic carbon photo-oxidation extend far beyond the canonical values used to determine gross photosynthesis. Consideration of these newly measured isotopic fingerprints suggests that gross photosynthesis may be significantly over- or underestimated depending on the extent of photooxidation.

The production of molecular oxygen (O_2_) coupled with the consumption of carbon dioxide (CO_2_) by marine photosynthetic organisms has far reaching consequences for Earth’s climate and surface redox state. For this reason, quantifying gross primary productivity (GPP), gross oxygen production (GOP), and net metabolic state (i.e. net carbon-fixing or carbon-oxidizing) is crucial to understand biogeochemical cycling at all scales. However, these metrics are difficult to constrain in practice. Of all available analytical approaches, the triple-oxygen isotope (TOI) composition of marine dissolved O_2_ is widely recognized as the best integrator of oxygen cycling and estimator of GOP (1, 2).

A major obstacle hindering the determination of GPP and GOP (which are related by C:O stoichiometry of photosynthesis; we thus refer to these collectively as “gross productivity”) is that a large fraction of photosynthetic O_2_ is subsequently consumed by the very same photosynthetic organisms that produce it. These oxygen-consuming reactions include oxic respiration, photorespiration, light-dependent oxygen consumption (e.g. Mehler reaction), and extracellular superoxide production (3–6). Concentration measurements of various carbon- and oxygen-bearing reactants and products are typically only useful for determining net productivity. Oxygen isotope methods offer the opportunity to interrogate this complex system, and they exhibit several advantages over other commonly used methods for estimating gross productivity at large spatiotemporal scales. For example, the TOI method estimates in-situ primary productivity integrated over the timescale of O_2_ residence time in the surface ocean (typically weeks) and circumvents so-called “bottle effects” of short-term ship-board incubations (7).

The TOI method exploits the distinct ^17^O composition, reported as Δ′^17^O (where Δ′^17^O = δ′^17^O—λ_RL_ δ′Δ′^18^O; note that λ, the TOI slope, and *θ*, the mass law, have distinct, but related meanings; see the “Methods” section for isotope notation and definitions), of photosynthetic O_2_ relative to that of the troposphere (2). This method distills the marine oxygen cycle to three components: (i) photosynthetic oxygen production, which generates O_2_ with an isotopic composition similar to that of seawater (8); (ii) mixing with tropospheric oxygen, which drives O_2_ concentrations and isotopic compositions toward equilibrium with the lower atmosphere, and (iii) cellular respiration, which decreases O_2_ concentrations and preferentially consumes lighter O_2_ isotopologues (i.e. imparts an isotope effect).

In practice, the TOI composition of marine dissolved O_2_ is typically reported relative to tropospheric O_2_ with a TOI slope value of λ_RL_ = 0.518 (see the “Methods” section), which is thought to represent the fixed fractionation associated with microbial respiration. This implies that photosynthetic production increases Δ′^17^O, equilibration with the atmosphere drives Δ′^17^O close to zero, and respiration does not influence Δ′^17^O. The resulting Δ′^17^O value can thus be used in conjunction with measurements of gas exchange, or piston velocity, to directly calculate gross productivity (9). Other common methods for measuring gross productivity, including light/dark bottle incubations and fast repetitive rate fluorometry, offer estimates on shorter timescales and over more limited conditions, which, in turn, leads to greater extrapolated uncertainty (10).

Uniquely determining gross productivity using the TOI method hinges on two central assumptions: (i) that the ^18^O and ^17^O fractionation of respiration (reported as ^18^α and λ, respectively, see the “Methods” section) are constant over space, time, environment, and ecology, and (ii) that respiratory oxygen utilization by microorganisms is the dominant sink of marine dissolved O_2_. While these simplifications conveniently reduce the interpretation of marine dissolved O_2_ Δ′^17^O values to a two end-member mixing problem between photosynthetic production and atmospheric gas exchange, the size, relative contribution, and TOI systematics of respiratory and nonrespiratory oxygen fluxes remain uncertain. This limits the precision and accuracy with which marine gross productivity can be approximated. For example, a recent biogeochemical study demonstrated that our current isotopic formulation of biospheric oxygen cycling is unable to reproduce tropospheric Δ′^17^O values (11). Specifically, this study demonstrated that newly determined respiration fractionation factors presented in Ash et al. (12) were unable to achieve mass balance in the global O_2_ isotope model presented in Young et al. (13). This discrepancy would suggest that (i) the average λ value assumed for biospheric respiration is too high, (ii) the method is missing an additional oxygen utilization pathway (with a lower λ), or (iii) a combination of both. A closer examination of the two foundational assumptions of TOI-derived productivity estimates is thus warranted to reconcile these theoretical and observational constraints on oxygen biogeochemistry.

In addition to revisiting the isotopic consequences of respiration on marine dissolved O_2_, here, we also consider an important nonbiological oxygen flux in the ocean. Light-dependent O_2_ consumption via photo-oxidation of dissolved organic carbon (DOC) has long been understood as quantitatively important in the photic zone of the marine water column (14–19). For example, photo-oxidation rates have been reported to exceed microbial respiration in the Mauritanian upwelling (17) and subtropical Atlantic surface waters (16). Moreover, assuming that one mol of O_2_ is photochemically consumed per mol of CO_2_ produced (15), photo-oxidation of exported terrestrial DOC accounts for at least 10% of total water-column oxygen consumption on the continental shelf of the Gulf of Mexico (20, 21). This estimation is likely conservative given that the ratio of photochemical O_2_ consumption to CO_2_ production is typically greater than one (22, 23). Despite this evidence that photochemical O_2_-consuming reactions are widespread in the surface ocean, and that photo-oxidation of organic carbon does preferentially consume lighter isotopologues of O_2_ (24, 25), this process has yet to be incorporated into TOI-measures of productivity (17).

To fill these critical knowledge gaps and better quantify marine oxygen cycling and gross productivity, here, we calibrate TOI fractionation generated by marine heterotrophic organisms and by DOC photo-oxidation. Specifically, we measure TOI signatures of O_2_ utilization from two model marine heterotrophic bacteria (*Vibrio harveyi* and *Ruegeria pomeroyi* DSS-3) and from photo-oxidation of terrestrial (Suwannee River, GA, USA) and coastal marine DOC (Martha’s Vineyard Sound, MA, USA). Using our experimental findings, we conduct a sensitivity analysis to interrogate how these new constraints and other systematic biases (e.g. the choice of a tropospheric O_2_ standard) influence TOI-derived productivity estimates. We conclude that TOI-based productivity methods can produce more than 20% over- or underestimation depending on the extent of photooxidation, type of DOC, and choice of tropospheric O_2_ reference value.

## Results and discussion

### Oxygen isotope fractionation by marine heterotrophs

**Fig. 1.**
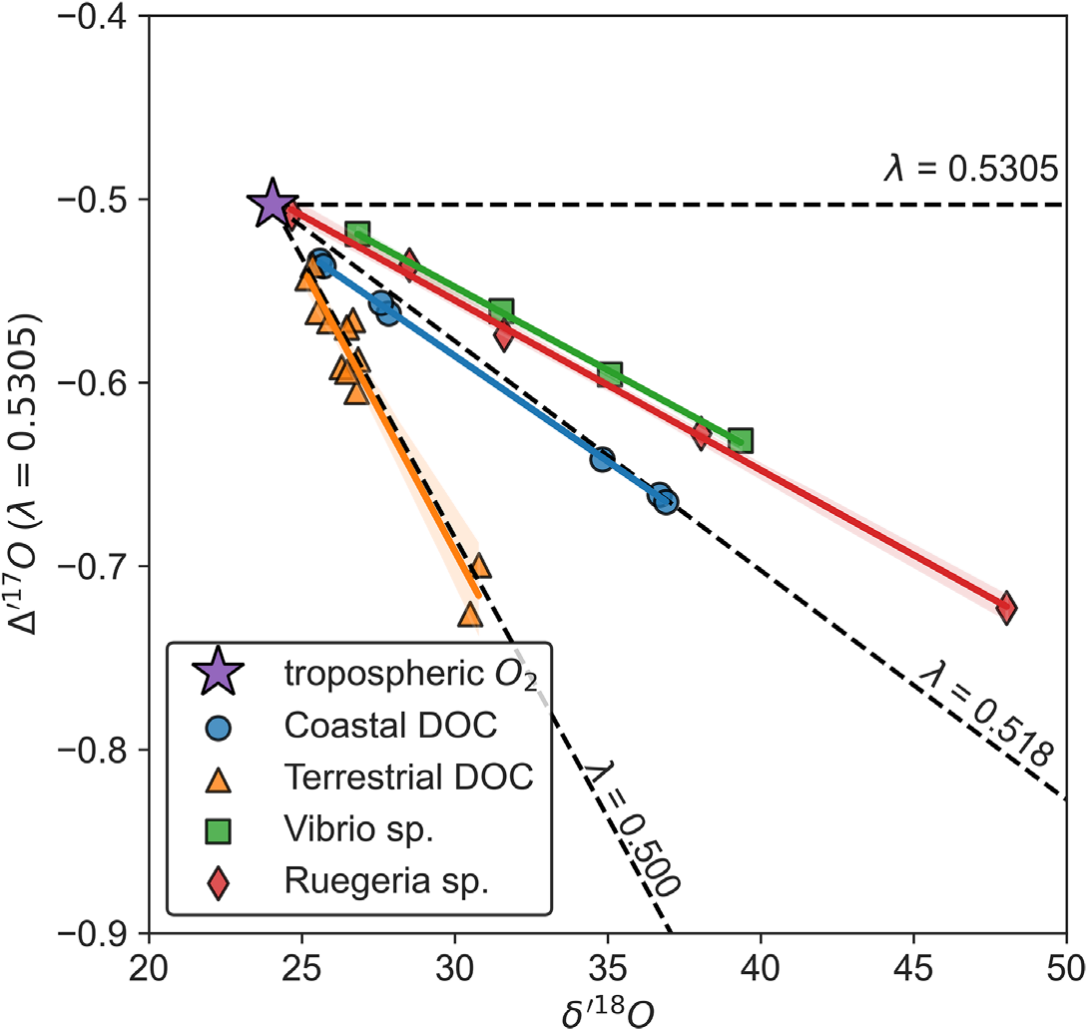
Three isotope plot of the evolution of residual dissolved O_2_ from biological and photochemical oxygen consumption. Values are plotted in linearized delta notation after Miller et al. (33) using a λ = 0.5305 reference line. These data include O_2_ utilization from *V. harveyi* (green), *R. pomeroyi* DSS-3 (red), photo-oxidation of Suwanee River DOC (labeled “Terrestrial DOC,” orange), and photo-oxidation of coastal DOC (blue). Best-fit lines were determined using ordinary least squares, and shaded region around each line represents 95% CI. For visual reference, we have also included dashed lines corresponding to λ = 0.5305, the reference slope and theoretical high temperature limit, λ = 0.518, the canonical respiration slope that we question in the present study, and λ = 0.500, an arbitrary low-end reference line. The slope produced by photochemical reactions deviates significantly from that of biological O_2_ utilization, suggesting aqueous environments in which both types of reactions are occurring will yield intermediate slopes. The tropospheric O_2_ value was taken as that reported in Wostbrock et al. (46).

Despite the continued addition of new experimental constraints (12, 26, 27), the TOI effect of marine heterotrophic bacteria—the most relevant ecological niche for O_2_ consumption in the global ocean—has yet to be determined. To address this, we measured oxygen isotope fractionation by two marine heterotrophic organisms—the Gammaproteobacterium *V. harveyi* and the Alphaproteobacterium *R. pomeroyi* DSS-3—both grown in batch culture under dark conditions (see the “Methods” section). Specifically, we measured isotopic compositions of residual dissolved O_2_ with cells respiring in midexponential growth. The average O_2_ consumption rate ranged from 12 to 18 μM O_2_ hour^−1^ across all incubations, with resulting O_2_ concentrations ranging from near saturation to a minimum of 26% saturation. Assuming Rayleigh fractionation (and without forcing regressions through a particular starting value; see the “Methods” section), we calculate that these organisms consumed O_2_ with ^18^α = 0.988 ± 0.004 and λ = 0.5214 ± 0.0004 (θ_EFF_ = 0.5199 ± 0.0004; effective θ assuming fractionation represents a single process) for *V. harveyi* and ^18^α = 0.9801 ± 0.0003 and λ = 0.5213 ± 0.0003 (θ_EFF_ = 0.5188 ± 0.0003) for *R. pomeroyi* (Fig. 1, Table 1, individual points are provided in the Supplementary Material). Rayleigh fractionation describes our measured isotope effects with a root-mean square error near that of analytical precision (RMSE = 0.002‰ and 0.004‰ for *Vibrio* and *Ruegueria*, respectively), implying that fractionation factors are independent of rate across our experimental conditions. The range of ^18^α values observed here is largely consistent with those reported previously; however, our λ values are notably higher than those used for respiration in gross productivity estimates (i.e. λ = 0.517 to 0.518; equivalent to λ_RL_ in the Δ′^17^O definition). We also note that the mass laws presented here for model marine Alpha- and Gammaproteobacteria are statistically indistinguishable from those recently reported for a natural lake sample, supporting a growing body of observational and theoretical work suggesting that the canonical λ values used for respiration should be revised upward (12, 28).

**Table 1.** Summary of measured TOI effects.

**O_2_-consuming reaction**	**^18^α ± 1σ**	**λ ± 1σ**	**θ_EFF_ ± 1σ**
*Heterotrophic Respiration*			
*Vibrio* sp.	0.988 ± 0.004	0.5214 ± 0.0004	0.5199 ± 0.0004
*Ruegeria* sp.	0.9801 ± 0.0003	0.5213 ± 0.0003	0.5188 ± 0.0003
*Photochemical Oxidation*			
Terrestrial DOC	0.991 ± 0.001	0.4994 ± 0.0026	0.4993 ± 0.0026
Coastal DOC	0.978 ± 0.002	0.5190 ± 0.0001	0.5162 ± 0.0001

There are several analytical and experimental explanations that could cause the range of observed heterotroph λ values. Analytically, varying O_2_: Ar ratios in sample gas can introduce a scale distortion in the mass spectrometer that requires a pressure baseline correction (12, 29). In the present study, Ar was cryogenically removed prior to analysis and is therefore not a concern (see the “Methods” section). Experimentally, previous studies have investigated O_2_ utilization in plants, animals, nonmarine bacteria (including *Escherichia coli*), and freshwater, and marine phototrophs. Multiple factors can determine oxygen isotope fractionation during respiration, including the physical transport to the binding site, the energetics of the O_2_ binding environment at the terminal O_2_ reductase, and subsequential electron transfer steps (12, 27). Additional aspects can also influence the expressed isotope effects, including complex tissue structure in animals, the activity of alternative oxidases, and nonrespiratory O_2_ sinks (i.e. other oxidoreductase enzymes throughout the cell). Perhaps most puzzling is the previously reported expression of λ values as low as 0.510 in wild-type *E. coli* (27)—a result yet to be observed in other microorganisms. Whatever complexity may exist, the internal consistency between the TOI effect of our two model marine heterotrophs and that of a natural consortium of lake bacteria in Ash et al. (12) lends support to the application of the TOI approach with a fixed λ for respiration.

One recent analysis suggested that these relatively low λ values observed in *E. coli* may arise from the production and cycling of reactive oxygen species resulting from nonrespiratory oxidoreductase enzymes (30). If this is correct, isotope effects typically assumed to represent “respiration” may truly represent a combination of respiratory and nonrespiratory O_2_ consumption. Although the λ values associated with reactive oxygen species production have not yet been directly observed, the excitation of colored DOC by light is known to be an important source of reactive oxygen to the surface ocean (31). Therefore, the exploration of DOC photo-oxidation in the following section may offer insight into the range of isotope effects for reaction pathways involving various forms of reactive oxygen.

### Oxygen fractionation by photo-oxidation of DOC

We provide the first triple-oxygen constraints on DOC photo-oxidation, a reaction whose oxygen demand can rival and even exceed that of biological processes in productive marine surface waters (15–17). The photo-oxidation of DOC will only have a notable impact on the isotopic composition of dissolved O_2_—and therefore gross productivity estimates—if its flux is quantitatively appreciable and its TOI slope(s) deviate significantly from that of microbial O_2_ utilization. In an effort to constrain the TOI effect of DOC photo-oxidation, we conducted photochemical incubations of terrestrial (Suwannee River, see the “Methods” section) and coastal marine (Vineyard Sound, see the “Methods” section) DOC under UVA light (369 ± 16 nm, see the “Methods” section) lasting between 3 and 107 hours.

Average O_2_ utilization rates ranged from 3 to 15  μM O_2_ hour^−1^ for terrestrial DOC and 1 to 4  μM O_2_ hour^−1^ for coastal DOC. Resulting TOI slopes and mass laws associated with O_2_ consumption from DOC photo-oxidation are notably lower than those reported for microbial O_2_ utilization. Photo-oxidation of terrestrial DOC exhibited ^18^α = 0.991 ± 0.001 and λ = 0.4994 ± 0.0026 (θ_EFF_ = 0.4993 ± 0.0026), whereas coastal DOC yielded ^18^α = 0.978 ± 0.002 and λ = 0.5190 ± 0.0001 (θ_EFF_ = 0.5162 ± 0.0001) (Fig. 1, Table 1, individual points are provided in the Supplementary Material). Interestingly, although coastal DOC photo-oxidation is well-described by this λ value (i.e. small uncertainty), the TOI array does not regress through the initial O_2_ composition. In fact, coastal DOC photo-oxidation is the only experimental condition whose 3 SD regression uncertainty does not encompass the starting dissolved O_2_ value. This can be accommodated, however, if λ is not static. For example, a regression between the starting dissolved O_2_ composition and first cluster of points (i.e. the two samples corresponding to the shortest incubation period) would produce a slope of 0.513 ± 0.002 before increasing to ∼0.519 for subsequent time intervals (not including starting point).

Given the complexity of photo-oxidation reactions, there are a number of potential explanations for this isotopic behavior—both within and between experiments. For example, the expression of an isotope effect is often sensitive to the degree of reversibility in a multistep pathway (e.g. the reversible formation of some excited state such as singlet oxygen, followed by an irreversible electron transfer step), which may change over the course of the reaction as the more labile components are consumed (32). Alternatively, there may be multiple light-reactive moieties with varying quantum efficiencies, consistent with observed decreases in quantum efficiency with increasing light exposure in coastal waters (i.e. photo-dose dependence) (15, 33). In both cases, photo-oxidation pathways and rates evolve as DOC is degraded and transformed into new compounds with differing chemical compositions and labilities to photo-oxidation. Whatever the case, it is clear that the photo-oxidation of marine DOC generates an isotope effect that differs from that of oxic respiration determined in this study, and can quantitatively impact gross productivity estimates.

In contrast to coastal DOC, the observed terrestrial DOC photo-oxidation TOI slope is among the lowest ever observed. The only precedent for such a low λ value is that of O_2_ consumption by the Mehler/Mehler-like reactions (i.e. light-dependent O_2_ reduction) in a cyanobacterial culture (5). The mechanisms of the Mehler/Mehler-like reactions are complex and not entirely resolved. The Mehler-like reaction in cyanobacteria is mediated by flavodiiron proteins that completely reduce O_2_ to water during light reactions (5). The Mehler reaction, which occurs in higher plants and in cyanobacteria, in addition to the Mehler-like reaction, is mediated by ferredoxin in photosystem I that reduces O_2_ to superoxide (6, 34, 35). The theoretical basis for the low λ value associated with the Mehler/Mehler-like reaction in cyanobacteria is not yet known, but given the similarity to photo-oxidation λ values observed here, this may relate to the formation of reactive oxygen species such as singlet O_2_ and/or superoxide. We note that previous studies have theoretically determined that the mass law associated with superoxide formation are notably higher than those observed for photo-oxidation (0.523 to 0.528 at surface temperatures), but direct measurement of this isotope effect is lacking (36). Alternatively, it is possible that the low λ value previously reported for the Mehler/Mehler-like reaction results from photo-oxidation of biomass or media constituents during light incubations (19). Field and modeling studies have invoked the seemingly exotic mass law of the Mehler reaction to explain significant TOI deviations in aqueous environments and to resolve mass balance in the troposphere (11, 37). Since the conditions under which the Mehler/Mehler-like reaction and photo-oxidation of DOC are almost entirely overlapping (i.e. sunlit, surface ocean), it is possible that both processes contribute to the expression of this noncanonical TOI slopes in the environment.

The simplest explanation for distinct TOI slopes between terrestrial and coastal DOC photo-oxidation is that the rate limiting step depends on chemical composition. This interpretation is consistent with well-documented differences in chemical composition and quantum efficiencies between terrestrial and marine DOC sources (15, 38, 39). The photochemical pathways that control DOC photochemical O_2_ consumption, and presumably the associated TOI slopes and their associated mass laws, are poorly understood for any DOC source. Initial reports suggested that up to 45% of photochemical O_2_ consumption by terrestrial and coastal DOC is mediated by hydrogen peroxide, with singlet oxygen (^1^O_2_) playing a minor role (i.e. <1% of O_2_ consumption) (15). Subsequent studies suggest a more prominent role of ^1^O_2_ for terrestrial and microbially derived DOC (i.e. up to 70% of photochemical O_2_ consumption) (32), particularly when considering the orders-of-magnitude higher concentrations of ^1^O_2_ detected in the hydrophobic interiors of DOC (40, 41). Similar heterogeneous radical production was recently reported for hydroxyl radical (42), renewing interest in the intermediate’s role in DOC photochemical oxidation. Despite sustained interest within the aquatic photochemical community, the governing pathway(s) of DOC photo-oxidation remains a gap in knowledge, in large part due to the challenges (e.g. specificity and observability) of using chemical probes to characterize different oxidative pathways (40–42). The distinct photochemical mass laws between DOC sources observed in this study thus create an opportunity to use oxygen isotopes, in addition to chemical probes, to better understand the importance of different forms of reactive oxygen, address long-standing gaps in knowledge about the governing pathway(s) of DOC photochemical O_2_ consumption, and investigate how the dominant photo-oxidation pathway may vary across DOC sources and sunlight exposure histories.

### Consequences for productivity estimates

Real-world gross productivity estimates are extremely sensitive to the chosen λ value for marine heterotrophy (i.e. λ_RL_). Specifically, if the applied λ_RL_ deviates from the true environmental net O_2_ consumption λ value (which integrates all O_2_ consuming processes from the microbial community and abiotic reactions), then O_2_ consumption will influence the Δ′^17^O and artificially increase or decrease corresponding gross productivity estimates. Here, we explore the potential impact on gross productivity estimates of two mechanisms that can influence λ_RL_: (i) an updated understanding of marine heterotrophy λ values, and (ii) nonmicrobial O_2_ consumption via photo-oxidation.

It has been a long-standing assumption that λ is invariant across nearly all O_2_-respiring organisms with a value of ∼0.518 (26). More recent experimental work expanded the range of observed respiration λ values from 0.510 to 0.523 (12, 27). For reference, λ variability on the scale of 0.001 leads to 10% to 15% error in calculated gross productivity (12), meaning that the potential difference in productivity estimates corresponding to this range of λ values is greater than a factor of two (5, 12, 26, 27, 43). Focusing just on the fractionation imparted by marine heterotrophs, revising λ values from the canonical ∼0.518 to the value we observed for marine heterotrophs (λ = 0.5214) suggests that current oxygen based gross productivity estimates are as much as 40% too high.

**Fig. 2.**
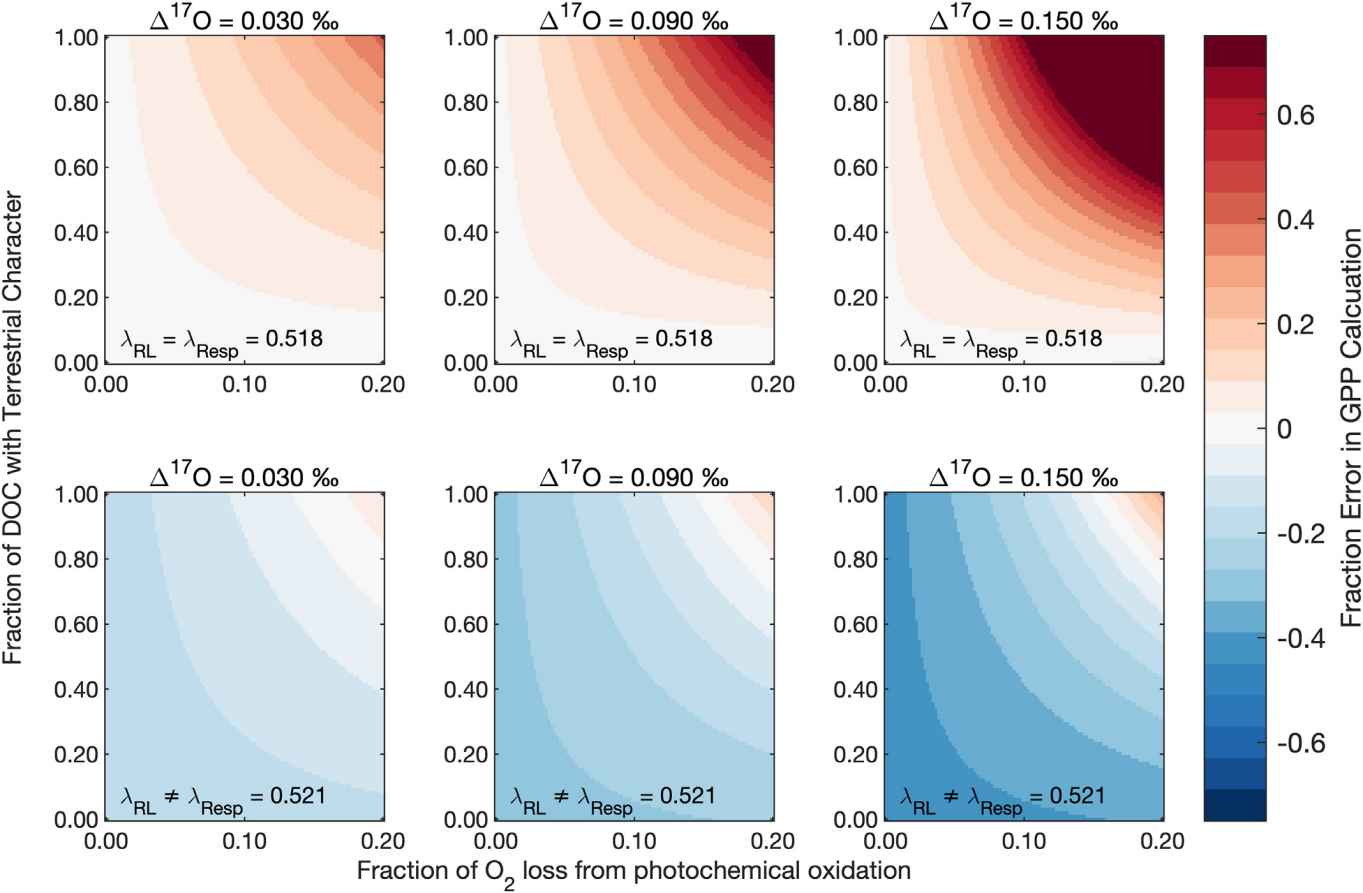
Difference between true and calculated gross O_2_ production for a range of possible water column Δ′^17^O values and photochemical contributions (both amount and fraction marine vs. terrestrial). The three panels in the top row assume that the commonly used λ_RL_ represents the true slope associated with dark microbial O_2_ utilization, while the three panels on the bottom row assume that the true slope of dark microbial O_2_ utilization is that reported in this study and in Ash et al. (12). Here, we conclude that λ = 0.521 for two model marine heterotrophic bacteria, meaning the images on the bottom row are more representative of current gross productivity bias.

Here, we assess the impact of our newly measured respiration and photo-oxidation λ values on gross productivity. A sensitivity analysis (see the “Methods” section) reveals that DOC source, the ratio of photochemical to biological O_2_ utilization, and the assumed λ value for microbial O_2_ utilization substantially impact TOI-derived gross productivity estimates (Fig. 2). For a fixed ratio of photochemical to biological O_2_ utilization, the most significant deviation from canonical λ_RL_ is likely to occur in near-shore environments where DOC concentrations, surface ocean productivity, and terrestrial organic matter runoff fluxes are all elevated. This is supported by Howard et al. (37), who recently used the TOI method to estimate gross productivity in a shallow salt marsh pond, exactly where terrestrial DOC photo-oxidation is expected to be maximized. The authors concluded that light-dependent biological oxygen consumption (e.g. Mehler reaction) leads to a significant disparity between TOI-derived gross productivity and O_2_-concentration derived estimates, with TOI-derived estimates exceeding concentration-derived estimates by 23% to 62%. Our findings suggest that the observations in Howard et al. (37) could be similarly explained by a significant contribution of photo-oxidation of terrestrial DOC to oxygen consumption.

Our sensitivity analysis indicates that this suite of respiration and photo-oxidation reactions exhibits diverging influences on the gross productivity bias, with respiration and photo-oxidation of coastal DOC biasing toward overestimation, and photo-oxidation of predominately terrestrial DOC biasing toward underestimation relative to current conventions. Even a modest contribution of photo-oxidation to net O_2_ consumption (e.g. 10%, without considering the impact of revising the respiration λ) will yield a gross productivity underestimation of ∼15% for a 50–50 mixture of coastal and terrestrial DOC and a Δ′^17^O signal of + 0.090‰ (note that typical photic-zone Δ′^17^O values range from + 0.010‰ to + 0.150‰ for low- and high-productivity depths, respectively, Fig. 2). This underestimation bias increases to >30% for the same conditions if the DOC pool is predominantly terrestrial in character. Furthermore, revising respiration λ values (to 0.521 from 0.518) will lower gross productivity estimates by 20% to 40% (Fig. 2). The magnitude of the corresponding gross productivity overestimation, therefore, is ultimately lessened by the co-occurrence of microbial oxidation and photo-oxidation. An upward revision of the respiration λ value we determine in this study makes TOI-based gross productivity values more sensitive to influence by photochemical processes. While ultimately choosing a single, representative value for λ_RL_ is convenient for readily relating TOI values to gross productivity, it is clear from this study that no such universal value exists. Rather, sunlit surface waters with high productivity will likely be dominated by a λ_RL_ that is less than 0.521 by some amount corresponding the amount and type of DOC. As depth increases in the water column, λ_RL_ would presumably approach 0.521 as photo-active wavelength(s) are attenuated.

**Fig. 3.**
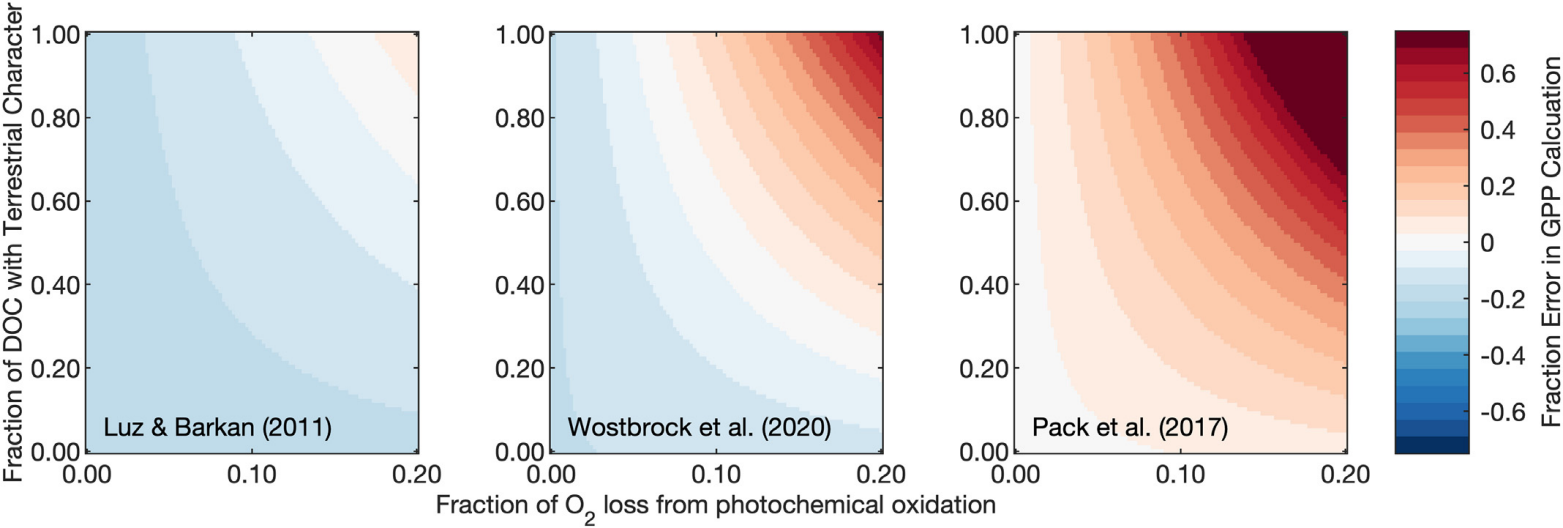
Difference between true and calculated gross O_2_ production for dissolved O_2_ with Δ′^17^O of + 0.030‰ assuming the three different reported values for tropospheric O_2_ isotope composition reported in Luz and Barkan, Wostbrock et al., and Pack et al. (44–46). Here, we assume that λ of respiration is 0.521. Note that the left panel is identical to the bottom left panel in Fig. 2, and all unstated assumptions are otherwise the same as those in Fig. 2.

These observations highlight a significant systematic error in gross productivity measurements that, when accounted for, can improve our understanding of the magnitude of Earth’s marine biosphere. It is important to point out that there are additional sources of systematic bias beyond that of respiration oxygen isotope fractionation. The uncertainty in the isotopic composition of the photosynthetic endmember, and more generally, the uncertainty in the reported value of the tropospheric O_2_ standard relatively to the Vienna Standard Mean Ocean Water 2 (VSMOW2) standard, carries with it bias that can be comparable in magnitude to that of photooxidation of DOC. As pointed out by Ash et al. (12), recent updates to tropospheric O_2_ isotope standards (e.g. Pack et al.) can potentially counteract the systematic bias resulting from using a respiration λ of 0.518 (44). We included this potentially significant bias in our sensitivity analysis (Fig. 3). Specifically, we explored the potential influence of assuming tropospheric O_2_ values reported in Luz and Barkan, Wostbrock et al., and Pack et al. (44–46). We find that assuming the tropospheric isotope values in Luz and Barkan and Wostbrock et al. leads to an underestimation of gross productivity as a result of revising the respiration slope (15% to 20% underestimation for a Δ′^17^O signal of + 0.030‰), while using the values in Pack et al. leads to near cancellation of errors with the revised respiration slope (<1% using the same assumptions) (44–46). Interestingly, while assuming the tropospheric O_2_ values from either Wostbrock et al. (46) and Pack et al. (44) decreases the net bias from these combined effects, the smaller Δ′^17^O offset between VSMOW2 and tropospheric O_2_ [compared to that in Luz and Barkan (45)] makes the overall gross productivity estimate more sensitive to photochemical oxygen consumption. Given that the standard calibration reported in Wostbrock et al. (46) is the most recent and comprehensive such standard calibration, and that the tropospheric O_2_ value reported in that study is near the mean of these three studies, the values reported in Wostbrock et al. (46) should serve as the basis for future gross productivity estimates until future revisions and/or inter-laboratory calibrations are conducted.

In addition to uncertainty in the isotopic endmembers, additional sources of uncertainty include: (i) piston velocity, (ii) mixed layer depth and its temporal evolution in response to environmental factors, (iii) isotopic variability of new photosynthetic O_2_ production, (iv) fractionation associated with O_2_ dissolution in seawater, and (v) extent to which other biogeochemical processes involving oxygen contribute to marine O_2_ cycling (9, 12, 30, 47, 48). Some of these terms, notably those related to oxygen isotope parameters, may represent systematic bias comparable in magnitude to that associated with the isotope effects of O_2_ utilization reported herein. Future work can further address systematic bias by refining the isotope systematics of O_2_ solubility, the scope of oxygen cycle reactions in the marine biogeochemical system, and the isotopic endmember(s) of photosynthetic production.

## Conclusion and future directions

We demonstrate that TOI slopes associated with O_2_ consumption by heterotrophic respiration and photo-oxidation of DOC span a wide range of the mass-dependent domain (λ = 0.4994 ± 0.0026 to λ = 0.5214 ± 0.0004). The λ values associated with two marine heterotrophs are internally consistent but differ substantially from the λ value commonly assumed for TOI applications. These new data also agree with more recent observations of dark O_2_ consumption by a natural freshwater microbial community (12, 26, 27). Dissolved O_2_ consumption from DOC photo-oxidation revealed a wide range of λ values, with terrestrial DOC exhibiting a very distinct TOI slope (λ = 0.4994 ± 0.0026). Photo-oxidation of coastal DOC exhibited λ = 0.5190 ± 0.0001; however, this process could exhibit values ≤0.513 in the initial stages of photo-oxidation. Although microbial oxygen consumption likely dominates integrated O_2_ consumption in the photic zone, the low λ values associated with DOC photo-oxidation underscore the significant leverage of this process on the TOI composition of marine dissolved O_2_. A sensitivity analysis revealed that even under moderate productivity and photo-oxidation scenarios, true gross productivity may deviate from traditional calculations by more than 20%. Additionally, the low λ values associated with DOC photo-oxidation likely play a role in reconciling the discrepancy between observed and theoretically determined tropospheric Δ′^17^O (11).

Through this work, we quantified the variability associated with key assumptions in the TOI method. Although this study focuses primarily on improving modern marine productivity estimates, similar isotope systematics are often applied to paleoenvironmental records of tropospheric O_2_ and productivity, including mineral-bound oxygen in sulfate and O_2_ gas trapped in ice cores (49, 50). We see several paths forward for improving TOI-derived gross productivity estimates, both in the modern marine environment and in its application to paleoenvironmental records. First, λ values arising from microbial O_2_ utilization should be characterized across a broader range of marine microbes (e.g. cyanobacteria, eukaryotic phytoplankton, heterotrophic bacteria abundant in oligotrophic waters, and representatives of other oxygen-consuming metabolisms that are relevant to the marine water column, such as nitrifiers). Second, the ratio of photochemical to biological O_2_ utilization should be properly constrained across space and time. This includes understanding how photochemical reactivity and TOI fractionation vary as a function of sunlight wavelengths (i.e. UVB to visible light regions) throughout the water column (15, 51). Third, λ resulting from photo-oxidation should be characterized across a broader range of DOC sources and functional group characteristics. Lastly, nonrespiratory O_2_-consuming reactions including the Mehler reaction, photorespiration, extracellular enzymatic degradation of marine DOC (52), and extracellular superoxide production (4, 53)—all potentially important oxygen fluxes with limited isotopic characterization—should be addressed in future studies. Filling these knowledge gaps will improve our understanding of oxygen and carbon fluxes in past and present marine environments, substantially reduce uncertainty in TOI-derived productivity estimates, and ultimately improve our ability to assess the metabolic state of marine environments and their response to global environmental change.

## Methods

### Oxygen isotope notation

Oxygen isotope compositions are typically written in δ notation, which expresses the ^17^O/^16^O or ^18^O/^16^O isotope ratio of a sample relative to a standard as

$$\begin{equation*}
\delta \ {}_{}^xO = \ \frac{{{}_{}^x{R}_{sample}}}{{{}_{}^x{R}_{standard}}} - 1,
\end{equation*}
$$

where *^x^R* is the atomic ratio of ^17^O or ^18^O relative to ^16^O. Values are typically reported in units of “permil” (‰) relative to VSMOW2 or modern tropospheric O_2_ standard reference material. For any kinetic or equilibrium chemical reaction (e.g. photosynthesis, respiration, photo-oxidation, etc.), the isotopic compositions of the reactant and product compounds are related by

$$\begin{equation*}
{}_{}^x\alpha \ = \frac{{{}_{}^x{R}_{product}}}{{{}_{}^x{R}_{reactant}}},
\end{equation*}
$$

where ^*x*^α is termed the isotope “fractionation factor.” For a specific reaction or process, the fractionation relationship between the two minor oxygen isotopes follows a mass law *θ* such that

$$\begin{equation*}
{}_{}^{17}\alpha = {\left( {{}_{}^{18}\alpha } \right)}^\theta .
\end{equation*}
$$

The TOI composition of dissolved O_2_ is typically reported in Δ′^17^O notation as

$$\begin{equation*}
{{\rm{\Delta ^{\prime}}}}^{17}O\ = {{\rm{\delta ^{\prime}}}}^{17}\ O - {\lambda }_{RL}{{\rm{\delta ^{\prime}}}}^{18}O,
\end{equation*}
$$

where

$$\begin{equation*}
{{\rm{\delta ^{\prime}}}}^xO\ = \ ln\left( {\frac{{{}_{}^x{R}_{sample}}}{{{}_{}^x{R}_{standard}}}} \right)
\end{equation*}
$$

is a linearized version of δ notation and λ_RL_ is a reference slope. In studies focusing on marine primary productivity, λ_RL_ is typically set to the empirically observed value describing dark, microbial oxygen utilization (0.516 to 0.518) (26). By setting the reference slope to be equal to the respiration slope, this practice results in respiration having no effect on reported Δ′^17^O values, thus reducing their interpretation to a two end-member mixing between tropospheric and photosynthetic O_2_. In other geochemical communities, reference slope (sometimes termed *θ*_RL_) values of 0.528 or 0.5305 are typically used to reference Δ′^17^O to the meteoric water line or the high-temperature equilibrium exchange limit, respectively. Given the uncertainty in λ corresponding to respiration and other empirical reference slopes, we present data relative to the 0.5305 reference line as it is a theoretical high temperature limit for λ_RL_.

We note that *θ* and λ have distinct uses in this isotope notation; *θ* is reserved to describe the fractionation relationship of a defined process or reaction (e.g. the fractionation relationship of an O_2_ reductase in respiration) whereas λ is an observed trend-line that may be the result of multiple factors (e.g. oxygen utilization in a closed or partially closed system such as the marine water column). In this study, we refer to λ as the TOI slope, and *θ* as the mass law. All λ values reported here were determined using a Rayleigh-distillation method, for which the relationship between *θ* and λ is defined in equation 17 of Angert et al. (26):

$$\begin{equation*}
\theta \ = \frac{{{\rm{ln}}\left( {1 + \lambda {}_{}^{18}\varepsilon } \right)}}{{{\rm{ln}}\left( {1 + {}_{}^{18}\varepsilon } \right)}},
\end{equation*}
$$

where ^*x*^𝜀 is defined as (^*x*^α–1). In the absence of a clear mechanistic understanding of the reactions producing the isotope effects in this study, we report the effective *θ* values as *θ*_EFF_ (i.e. the effective *θ* value assuming the fractionation is the result of a single, defined process).

### Microbial cultures

Cultures of *V. Harveyi* were grown in Tibbles-Rawlings minimal media and cultures of *R. Pomeroyi* DSS-3 were grown in K media as previously described (54, 55). Agar plates of each axenic culture were inoculated with a freezer stock and visually inspected for culture purity. A single colony from each plate was used to inoculate 50 mL of liquid media in Erlenmeyer flasks, which were incubated in the dark on an orbital shaker at 100 rpm. Cells were transferred fresh media during midexponential growth phase (as determined by optical density at 600 nm) for a minimum of two generations prior to incubation for analysis. For O_2_ utilization experiments, several mL of midexponential cell culture was aseptically added to several 500 mL serum vials, which were filled with fresh media (filter sterilized to avoid the production of O_2_-consuming byproducts, equilibrated overnight at room temperature on a stir plate to ensure oxygen isotopic equilibrium with lab air) then sealed with no headspace. The incubations were conducted at room temperatures, which ranges from 21 to 23°C. A typical incubation included between 4 and 6 bottles, which were sacrificially sampled at each timepoint. At the desired sampling time, sample liquid containing dissolved O_2_ was transferred to mercury-poisoned bottle as discussed in the analysis section below.

### DOC preparation and chemical characterization

DOC used in this study was sourced from the Suwannee River, Georgia and Martha’s Vineyard Sound, MA, USA. Suwannee River DOC was prepared from a freeze-dried reverse osmosis isolate provided by the International Humic Substance Society (#2R101N; 84% recovery) (56). The isolate was reconstituted in ultra-pure water (DI; 18.2 MΩ cm; Milli-Q IQ 7000; Millipore Sigma) in a 4 L precombusted amber glass jug at a concentration of 20 mg L^−1^ (9.7 ± 0.2 mg-C L^−1^; *n* = 3). The solutions were adjusted to pH 7.0 ± 0.1 and allowed to equilibrate with the atmosphere on a stir plate for 24 h prior to filtration with a 0.2 μm Sterivex filter (Millipore) and use in photochemical incubations.

Martha’s Vineyard Sound seawater was pumped from ∼300 m offshore (41.530668, −70.645629) at a depth of ∼4 m into the Environmental Systems Laboratory (ESL) at Woods Hole Oceanographic Institution (WHOI, Woods Hole, MA, USA). At high tide in June of 2021, ∼210 L of seawater was filtered through precleaned (200 L of RO and 100 L of DI water) 0.2 µm ultra-pleat in-line filters (Big Brand Water Filter, Inc.). The seawater DOC was collected in 12 five-gallon, acid-rinsed (10% trace metal grade hydrochloric acid; Fisher Scientific), and precleaned (10x rinses with RO and MQ water) polypropylene buckets (Uline, Inc.). The DOC was isolated using six 5 g PPL cartridges (Agilent Technologies) (57, 58). The methanol eluent from the six cartridges was pooled into one sample. DOC recovery was measured by spiking 100 µL of the pooled eluent into a precombusted TOC vial, drying over high-purity N_2_ (Airgas, Inc.), reconstituting in DI water, and quantifying using a Shimadzu 5000A TOC analyzer. DOC recovery was 44% ± 3% (*n* = 3). This recovery is ∼15% lower than reported for Brazilian coastal waters (57). This discrepancy was expected provided the 10× higher C-loading rate used in this study compared to Dittmar et al. (0.4 vs. 0.04 mmol-C g^−1^ PPL resin), and the well documented decline in DOC recovery with increased C-loading rates (59). Martha’s Vineyard Sound DOC SPE eluent was subsequently used to create concentrated solutions of coastal DOC (14.6 ± 0.2 mg-C L^−1^; *n* = 3). The eluent was added to a 4 L precombusted amber glass jug, dried over high-purity N_2_, and reconstituted in 0.2 µm filtered Martha’s Vineyard Sound seawater. The coastal DOC solutions were allowed to equilibrate with the atmosphere on a stir plate for 24 h prior to refiltration with a 0.2 μm Sterivex filter and use in photochemical incubations.

The working solutions of terrestrial and coastal DOC were chemically characterized using optical spectroscopy. Specifically, optical proxies for molecular weight and aromaticity were measured [E_2_: E_3_, (60); slope ratio (S_R_), (38); specific UV absorbance at 254 nm (SUVA_254_), (61)]. UV-visible light absorbance was determined using a Perkin Elmer Lambda 650 s spectrophotometer (51), whereas DOC concentration was determined as previously described. The optical proxy values determined in this study for terrestrial and coastal marine DOC strongly aligned with those reported in previous studies (38, 62, 63). The DOC sources were chemically distinct; terrestrial DOC exhibited notably higher molecular weight and aromaticity than coastal DOC (Table S1).

### Photochemical incubations

The working solutions of terrestrial and coastal DOC were transferred to 500 mL quartz bomb flasks equipped with 24/40 ground glass joints (Technical Glass Products, Inc.). The flasks were sealed with no headspace using UV resistant Perfluoropolyether grease (geminYe RT15, Inland Vacuum Industries, Inc.) and Keck taper clips (Cole-Parmer, Inc.). The flasks were placed underneath a custom-built 10 × 1 UVA LED chip array [369 ± 16 nm at full width at half maximum (FWHM), 2 W optical power per LED chip, 120° beam angle, LG 6060 series; (51)] powered by a 360 W power supply (# 2260B-250–4; Keithley). The peak wavelength and FWHM of the LED irradiance spectrum were determined using a NIST-calibrated spectroradiometer (Black Comet C-50; StellarNet, Inc.). Irradiance underneath the LED array was equivalent to 4 and 18 days of natural UVA light (integrated from 320 to 400 nm) at 40°N on June 21 and December 22, respectively (64). The array was cooled by a heat sink (# ATS-EXL312-300-R0; Advanced Thermal Solutions, Inc.) and two cooling fans (# Al-MPF120P2; AC Infinity). The temperature of the samples was 1 to 2°C warmer than room temperature, as determined using an IR thermometer (62 Max Plus; Fluke Corporation). Given the lower absorption of 369 nm light by coastal vs. terrestrial DOC (Fig. S1), longer light exposure times were required for the coastal DOC samples to ensure sufficient photochemical O_2_ uptake. The coastal DOC samples were exposed for up to 108 hours, whereas the terrestrial DOC samples were exposed for up to 24 hours. Dark controls were wrapped in aluminum foil and run alongside light-exposed treatments and exhibited no discernable change in O_2_ concentration or isotopic distributions. An additional set of negative controls consisting of 18.2 MΩ cm water was run to ensure that interactions between water and UVA light were not responsible for any of the isotope effects observed in this study. We found no statistically significant difference between irradiated water (*n* = 2) and dark controls (*n* = 2) after 120 hours of incubation. At the desired sampling times, light-exposed and dark-control samples were immediately prepared for TOI analysis as discussed below.

### TOI analysis

After each incubation (either microbial or photochemical), the seal on each closed bottle was opened and an O_2_ electrode was inserted for 15 seconds to get an approximate dissolved O_2_ concentration for later comparison with O_2_: Ar ratio to ensure no atmospheric contamination. Roughly half of sample liquid was then siphoned out from the bottom of the sample bottle into a preevacuated, prepoisoned (with 500 uL saturated HgCl_2_ solution) 1 L custom-built glass bottle affixed with a high-vacuum stopcock, similar to previous studies [e.g. (27)]. Method blanks were conducted with O_2_-free water (bubbled vigorously with helium for 1 hour) to ensure the above methods did not introduce atmospheric contamination. We found the method blank to be sufficiently low as to not influence oxygen isotope measurements within the precision reported in this study (*n* = 2). The sample bottle was vigorously shaken and allowed to equilibrate overnight. Following equilibration, the sample bottle was inverted, and the degassed sample liquid was evacuated, save 1 to 2 mL of residual liquid to ensure no sample gas was removed. The sample bottle was submerged in a slurry of dry ice and ethanol to freeze all remaining liquid, and the sample gas was introduced to a vacuum line for purification similar to that described in ref. (12). Oxygen gas was separated from N_2_ and Ar using a 3 m gas chromatography (GC) column packed with 5 Å molecular sieve held at −80°C. The integrated peak area (measured by thermal conductivity detector) of the separated O_2_ and Ar peaks was used to determine the fraction of the starting O_2_ that remained at each timepoint. The effluent O_2_ was collected on silica gel held at at −196 °C and passed to one final silica gel cryo-focus. The sample was then transferred to a Thermo Scientific MAT 253 Plus isotope ratio mass spectrometer (IRMS) at Harvard University (65).

Each reported analysis is the average value of four acquisition blocks, each consisting of 20 cycles between the reference gas and sample gas (total counting time on sample gas was 400 seconds per acquisition). Measurements were typically analyzed at 5000 mV signal on the m/z 32 Faraday cup (3 × 10^8^ Ω resistor). Total acquisition time for a single analysis is roughly 2 hours. All values of δ′^17^O and δ′^18^O were converted to a VSMOW2-SLAP2 scale using a two point calibration of O_2_ liberated form VSMOW2 and SLAP2 standards using a CoF_3_ reactor as previously described (66, 67). All slopes (i.e. λ values) and slope uncertainties were calculated as linear fits to plots of δ′^17^O vs. δ′^18^O using the polyfit function in MATLAB (linear least-squares). Typical reproducibility of Ar-free lab air is 0.005‰ for Δ′^17^O and 0.02 ‰for δ′^18^O (1σ, *n* = 10).

### Sensitivity analysis

To determine the sensitivity of GPP estimates to different values of λ, we calculated the theoretical λ for different combinations of microbial and photochemical O_2_ consumption. In this computation analysis, we allowed the photochemical O_2_ consumption to vary between 0% and 20% of total O_2_ consumption and the DOC character to vary between the coastal and terrestrial endmembers used in this study. Additionally, we explored cases in which we assumed the canonical respiration value (0.518) and the value determined in this study (0.521). The resulting λ was then used to calculate GPP as described in Prokopenko et al. (9). Lastly, the newly calculated productivity values were ratioed to the productivity estimate assuming no photochemical O_2_ demand and respiration λ of 0.518. We also explored the consequences of recently revisions of the reported TOI values of tropospheric O_2_ relative to the VSMOW2 standard. As pointed out in Ash et al. (12), the assumed reference value for air can introduce additional artifact to gross productivity comparable in magnitude to consideration of photochemistry and revision of the respiration slope. We therefore conducted the same sensitivity analysis described above for three reported values of tropospheric O_2_ (44–46).

## Supplementary Material

pgac233_Supplemental_Files

## Data Availability

All data are included in the manuscript and/or Supplementary Material.
