# Supplementary material for: Revised microbial and photochemical triple-oxygen isotope effects improve marine gross oxygen production estimates
Source: PNAS Nexus. 2022 Oct 12;1(5):pgac233. doi: 10.1093/pnasnexus/pgac233 (PMC9802178; doi:10.1093/pnasnexus/pgac233)
Supplement: pgac233_Supplemental_Files [file pgac233_supplemental_files.zip › PNASNEXUS-PNASNEXUS-2022-00672-T-s01.pdf]

Supplementary Information for
Revised Microbial and Photochemical Triple-Oxygen Isotope Effects Improve Marine Gross Oxygen Production Estimates

Kevin M. Sutherland[1,*], David T. Johnston[1], Jordon D. Hemingway[2], Scott D. Wankel[3], Collin P. Ward[3,*]

Affiliations:
[1]Department of Earth and Planetary Sciences, Harvard University, Cambridge, MA 02138, USA
[2] Geological Institute, Department of Earth Sciences, ETH Zürich, 8092 Zürich, Switzerland
[3]Department of Marine Chemistry and Geochemistry, Woods Hole Oceanographic Institution, Woods Hole, MA 02543

Correspondence to: Kevin M. Sutherland, Collin P. Ward
Email: ksutherland@fas.harvard.edu, cward@whoi.edu

This PDF file includes:

Figures S1
Tables S1

Other supplementary materials for this manuscript include the following:

S1 Data Table

Figure S1- Absorption spectra of terrestrial (blue) and coastal marine (orange) DOC samples used in photochemical incubations.

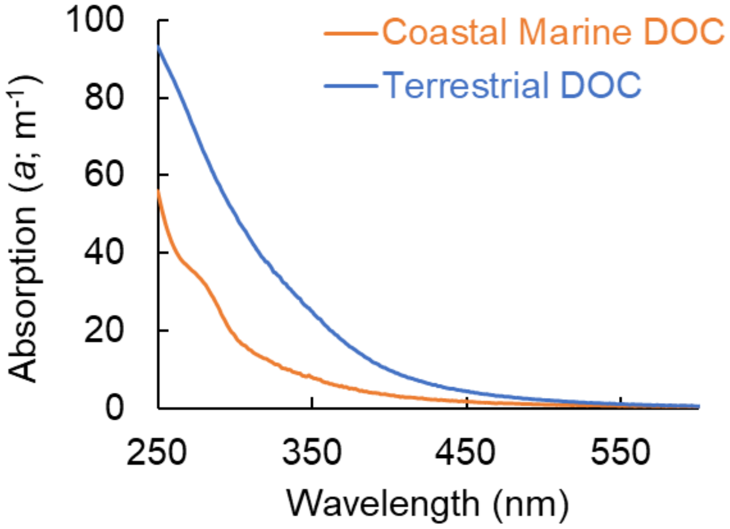

Table S1- Slope ratio (S_R) (38), E_2:E_3 (60), and specific UV absorbance at 254 nm ($SUVA_{254}$) (61). Error represents 1 standard deviation (n=3).

	**Slope Ratio**	**E_2:E_3**	**A_{254}**	**DOC**	**$SUVA_{254}$**
**DOC Source**	-	-	m^{-1}	$mg\text{-}C\ L^{-1}$	$L\ (mg\text{-}C\ m)^{-1}$
Terrestrial	0.76 ± 0.01	4.9 + 0.1	39.0 + 0.1	9.7 + 0.2	4.0 + 0.1
Coastal Marine	1.41 ± 0.04	9.7 + 0.6	21.2 + 0.2	14.6 + 0.2	1.4 + 0.1
